# Improved Healthcare Access Reduces Requirements for Surgery in Indigent IBD Patients Using Biologic Therapy: A ‘Safety-Net’ Hospital Experience

**DOI:** 10.3390/pathophysiology29030030

**Published:** 2022-07-18

**Authors:** Phillip Gu, Eric Clifford, Andrew Gilman, Christopher Chang, Elizabeth Moss, David I. Fudman, Phillip Kilgore, Urska Cvek, Marjan Trutschl, J. Steven Alexander, Ezra Burstein, Moheb Boktor

**Affiliations:** 1 Division of Digestive and Liver Diseases, UT Southwestern, Dallas, TX 75390, USA; phillipgu12@gmail.com (P.G.); andrew.gilman@utsouthwestern.edu (A.G.); david.fudman@utsouthwestern.edu (D.I.F.); ezra.burstein@utsouthwestern.edu (E.B.); moheb.boktor@utsouthwestern.edu (M.B.); 2 Department of Computer Science, Louisiana State University, Shreveport, LA 71103, USA; ecliff@lsu.edu (E.C.); pkilgor@lsus.edu (P.K.); mtrutsch@lsus.edu (M.T.); 3 Department of Medicine, UT Southwestern, Dallas, TX 75390, USA; christopher.chang@utsouthwestern.edu; 4 Ambulatory Care Pharmacy, Parkland Memorial Hospital, Dallas, TX 75390, USA; elizabeth.moss@utsouthwestern.edu (E.M.); ucvek@lsus.edu (U.C.); 5 Department of Molecular and Cellular Physiology, LSUHSC-S, Louisiana State University, Shreveport, LA 71103, USA

**Keywords:** Crohn’s disease, ulcerative colitis, inflammatory bowel disease, healthcare disparities

## Abstract

Low socioeconomic status (SES) is associated with greater morbidity and increased healthcare resource utilization (HRU) in IBD. We examined whether a financial assistance program (FAP) to improve healthcare access affected outcomes and HRU in a cohort of indigent IBD patients requiring biologics. IBD patients (>18 years) receiving care at a ‘safety-net’ hospital who initiated biologics as outpatients between 1 January 2010 and 1 January 2019 were included. Patients were divided by FAP status. Patients without FAP had Medicare, Medicaid, or commercial insurance. Primary outcomes were steroid-free clinical remission at 6 and 12 months. Secondary outcomes were surgery, hospitalization, and ED utilization. Multivariate logistic regression was used to calculate odds ratio (OR) and 95% confidence interval (CI). Decision tree analysis (DTA) was also performed. We included 204 patients with 258 new biologic prescriptions. FAP patients had less complex Crohn’s disease (50.7% vs. 70%, *p* = 0.033) than non-FAP patients. FAP records indicated fewer prior surgeries (19.6% vs. 38.4% *p* = 0.003). There were no statistically significant differences in remission rates, disease duration, or days between prescription and receipt of biologics. In multivariable logistic regression, adjusting for baseline demographics and disease severity variables, FAP patients were less likely to undergo surgery (OR: 0.28, 95% CI [0.08–0.91], *p* = 0.034). DTA suggests that imaging utilization may shed light on surgical differences. We found FAP enrollment was associated with fewer surgeries in a cohort of indigent IBD patients requiring biologics. Further studies are needed to identify interventions to address healthcare disparities in IBD.

## 1. Introduction

Inflammatory bowel diseases (IBD), include Crohn’s disease (CD), ulcerative colitis (UC), and indeterminate colitis, are immune-mediated GI disorders associated with significant morbidity and economic impacts. The current estimated annual cost of treating IBD is USD 6.3 billion in the US, and despite advancements, treatment costs continue to rise [1,2,3,4]. While surgery and hospitalizations were previously reported to be the primary factors explaining risings cost for IBD treatment, biologic agents have recently taken on a larger role [1,2,3,4,5,6]. Importantly, delays in providing treatment modalities that can suppress or arrest IBD disease activity can increase long-term morbidity while also increasing overall healthcare costs by creating more and serious complications. Consequently, strategies that accelerate delivery of healthcare resource utilization (HRU) in IBD are critically important and will likely improve patient outcomes.

Low socioeconomic status (SES) is a well-described risk factor for poor health outcomes and higher healthcare resource utilization in a number of chronic illnesses [7,8,9,10]. Interestingly, previous studies have found no association between disease severity, phenotype, or medication adherence and low SES in IBD patients that could explain differences in HRU and outcomes [11,12,13,14,15]. Thus, this suggests the possibility that low SES, by limiting and/or delaying access to critical resources needed for timely diagnosis and treatment, may result in more frequent, and potentially preventable, disease complications. Therefore, identifying interventions to improve healthcare access, such as access to medications, provider visits, procedures, and labs, in low SES patients with IBD may ultimately reduce IBD-related complications and improve the overall economic burden of IBD on the healthcare system.

To improve healthcare in the indigent patient population of Dallas County, TX, Parkland Memorial Hospital (PMH), a ‘safety-net’ hospital, have implemented a financial assistance program (FAP) to improve healthcare access for uninsured and underinsured Dallas County residents. The FAP reduces out-of-pocket costs for medications and medical services rendered at PMH. For IBD patients, this includes a significantly discounted cost and easier access (i.e., no insurance pre-authorizations) to obtain biologics, especially anti-TNF agents. While many prior studies have identified impaired access to care as a risk factor for poor outcomes, it is not clear whether improved healthcare access has an impact on outcomes and resource utilization in this unique IBD population. Because IBD patients requiring biologic agents are at highest risk for poor outcomes and increased resource utilization, we evaluated the impact of a FAP on healthcare outcomes and healthcare resource utilization in a cohort of indigent IBD patients requiring biologic agents.

## 2. Materials and Methods

### 2.1. Study Design and Patient Population

We performed a retrospective cohort study of patients with IBD seen in our gastroenterology clinics at PMH, which is a large safety net hospital system serving the residents of Dallas County, Texas. PMH is unique in that it is closed healthcare system, such that the majority of patients obtain all aspects of their care at PMH, including primary care, specialist care, surgical care, etc. IBD patients were identified through our electronic medical records using ICD 9 and 10 codes for Crohn’s disease (ICD-9 555, ICD-10 K50), ulcerative colitis (ICD-9 556, ICD 10 K51), and indeterminate colitis. No surgeries were studied beyond 12 months.

We included IBD patients aged >18 years who initiated a new biologic agent intended to treat their IBD between 1 January 2010 and 1 January 2019, with at least 3 months of outpatient follow-up at PMH. Patients were excluded if they had <3 months of follow-up or if the biologic was intended to treat a condition other than IBD. Outcomes after each new biologic prescription were analyzed separately. Included patients were dichotomized by FAP status. At PMH, the FAP provides financial assistance for medical services (i.e., medications, labs, procedures, outpatient visits, etc.) rendered at PMH for qualified individuals based on household income in relation to the Federal Poverty Income Level. Patients enrolled in the FAP were underinsured or uninsured. Non-FAP patients had insurance coverage with Medicare, Medicaid, or commercial insurance. FAP and non-FAP patients were equally eligible for all the same medical care at PMH. Patient outcomes were ascertained via manual chart review for up to 12 months of follow-up after starting a biologic agent. Manual chart review was also performed to obtain demographic and clinical data. Disease location and behavior were defined using the Montreal classification system [16]. Fibrostenosing and penetrating CD were combined in a composite group called “complex CD”. Although in some analyses we combined both UC and CD as ‘IBD’, we also evaluated data for both groups separately.

Patient-level analysis, as it relates to FAP status, was performed on data that was not likely to change between records (gender, race, IBD diagnosis, mean number of biologics, CD/UC location, complex CD behavior, perianal CD, upper GI involvement). Some of these measures had a minimal number of changes between records for patients, but for the sake of analysis, we use the patient’s first record.

Record-level analysis, as it relates to FAP status, was performed on data that was likely to change between records (age, BMI, smoker status, disease duration, active medications, prior surgeries, biologic naïve, number of clinic visits, time between prescription and initiation, medical outcomes). The Institutional Review Board at UT Southwestern and the Research Office at PMH approved this study (IRB number: 27569, 18 April 2019).

### 2.2. Outcomes

The primary outcome was corticosteroid-free clinical remission (CSFR) at 6 and 12 months after initiation of a biologic agent. Assessment for clinical remission was determined based on physician global assessment, where CSFR was defined as resolution of CD or UC-related symptoms without need for corticosteroids. The secondary outcomes included (i) IBD-related hospitalizations, (ii) IBD-related surgeries, (iii) emergency department and/or urgent care visits during the 12 months of follow up after initiating the biologic agent, and (iv) CT/MRI scans during the 12 months of follow up after initiating the biologic agent.

### 2.3. Statistical Analysis

For univariate analysis of relationships between data and FAP status, significance was determined using chi-square for categorical variables and binary logistic regression or Wilcoxon rank-sums test for continuous variables. Multivariate logistic regression was used to calculate odds ratio (OR) and 95% confidence interval (CI) for outcome variables with significant differences. Statistical significance was defined at *p* < 0.05.

The data was further analyzed using decision tree analysis (DTA). This was accomplished using the Conditional Inference Tree (ctree) functionality of the ‘partykit’ R library. This method uses decision tree induction to generate a decision tree that predicts the outcome in question. The first node, or root node, is the node that starts the decision algorithm and is the best variable that predicts/splits the data by the outcome. Factors used in the analysis include age, BMI, smoker status, disease duration, active medication, prior surgeries, number of clinic visits, prior surgeries, biologic-naïve status, race, weight, IBD diagnosis, PFA status, amount of time between prescription and initiation of biologic, and the number of CT/MRIs, ED visits, and hospitalizations within 12 months following initiation of the current biologic. Each split represents the value of only one variable, and DTA rejects variables that have no role in arriving at the variable in question or that are not statistically significant.

All statistical analyses were performed using the R statistical language (R Core Team. R: A language and environment for Statistical Computing. R Foundation for Statistical Computing, Vienna, Austria. 2017. URL: https://www.r-project.org (accessed on 12 May 2021)).

## 3. Results

### 3.1. Study Cohort

We included 204 unique patients with 258 new biologic prescriptions (records). The list of biologics we analyzed includes Adalimumab (Humira), Infliximab, Ustekinumab (Stelara), Vedolizumab (Entyvio), Golimumab, Certolizumab and Natalizumab. Comparison of baseline, record-level characteristics are summarized in Table 1. When comparing FAP and non-FAP records, there were no significant differences in active smoking status, age, BMI, disease duration, active medications, or biologic-naïve status. A significantly lower proportion of FAP records indicated previous IBD-related surgeries (19.6% vs. 38.4% *p* = 0.003). In looking at factors related to healthcare access, there was no difference in mean number of clinic visits (*p* = 0.942) or in mean number of days to receive a biologic agent after placement of the prescription (*p* = 0.104).

Comparison of baseline, patient-level characteristics are summarized in Table 2. Statistical significance was performed using chi-square, unless otherwise stated in the table. Statistics pertaining to Crohn’s Disease (location, complex behavior, perianal, and upper GI involvement) were only measured among the subset of patients with Crohn’s Disease. Similarly, statistics pertaining to Ulcerative Colitis (location) were only measured among UC patients.

These statistics pertain only to each patient’s first record. There were cases when some of these values may change between records: Differences in IBD Diagnosis were handled by including the patient in any group for which they were diagnosed. Thus, a patient whose first record is for UC, and second record is for CD, would be counted in both groups. This same procedure was used for CD location (two non-FAP, one FAP patients) and UC location (one FAP patient). Two patients (both FAP) had changing complex CD behavior designations, and one FAP patient had a change in perianal CD classification. These values are TRUE/FALSE values, so a change indicates a TRUE value at some point. As such, these patient were counted when a changing value occurred.

When comparing FAP and non-FAP patients (Table 2), there were no significant differences in IBD diagnosis, CD or UC location, upper GI involvement, or perianal CD status. Non-FAP patients were more likely to be female than FAP patients (59.4% vs. 42.1%, *p* = 0.032), as well as more likely to have complex CD behavior (in CD patients, 70% vs. 50.7%, *p* = 0.033). Significant differences among the two groups exist with respect to race (*p* ≤ 0.001): FAP patients were more likely to report as Hispanic (45.7% vs. 12.3%), while non-FAP patients were more likely to report as Black (54.7% vs. 25%). Finally, FAP patients had a higher mean number of biologics than non-FAP patients (1.32 vs. 1.14, *p* = 0.002).

### 3.2. Healthcare Outcomes

There was no significant difference between FAP and non-FAP patients in achieving CSFR at 6 months (38.2% vs. 34.4%, *p* = 0.845) and at 12 months (45.2% vs. 36%, *p* = 0.526, Table 3). In terms of secondary outcomes, there was no significant difference in mean number of emergency department (ED) visits (*p* = 0.789) and proportion of patients requiring IBD-related hospitalization (*p* = 0.590). A significantly lower proportion of FAP patients required surgery compared to non-FAP patients (5.6% vs. 16.7%, *p* = 0.034). Using a multivariate logistic regression model to adjust for age, gender, race, prior IBD-related surgery, disease duration, and complex CD behavior, FAP status was found to be independently associated with protection from surgery during the follow-up period after initiation of a biologic (odds ratio: 0.28, 95% confidence interval (CI) [0.08–0.91]). In a subgroup analysis of patients with a prior history of IBD-related surgery, FAP remained independently associated with less need for surgery (OR: 0.14, 95% CI [0.02–0.99]), *p* = 0.049).

**Figure 1:**
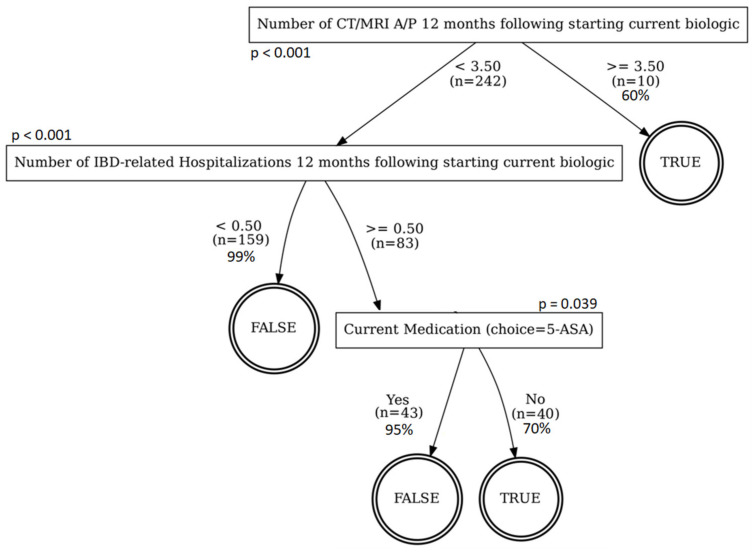
Decision-tree analysis of baseline demographic variables to predict surgery in IBD patients regardless of FAP enrollment. The percentage represents the accuracy, while n represents the number of patients that apply to a given step.

**Figure 2:**
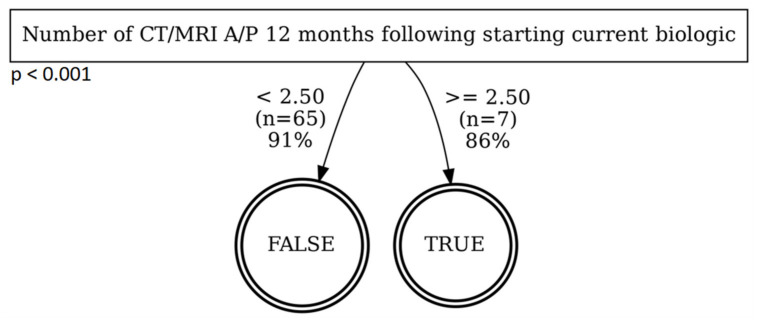
Decision-tree analysis of baseline demographic variables to predict surgery in IBD patients not enrolled in FAP.

In order to identify factors that may explain the differing surgical rates between FAP and non-FAP patients, we performed a decision tree analysis considering all baseline factors in Table 1, as well as race, weight, IBD diagnosis, PFA status, and the number of CT/MRI, ED visits, and hospitalizations within 12 months following initiation of the current biologic. These factors were used to determine which might predict or contribute to the need for surgery. Patients overall and non-FAP patients were analyzed separately (Figure 1 and Figure 2, respectively); the decision tree classifier for FAP patients unilaterally predicts no surgery with 94.475% accuracy; this outcome is attributable to the low number of FAP patients with surgery (*n* = 10) versus those who did not (*n* = 171). In the overall model, CT and MRI imaging of the abdomen/pelvis was the first ‘splitting’ variable, and a best cut-off level of 3.5 studies was identified. Sixty percent (out of *n* = 10) of patients who underwent >3.5 studies required surgery. The need for hospitalization was identified as the second splitting variable; 99% of patients could be correctly classified as not needing surgery. Finally, baseline 5-ASA use was the last splitting variable, with 52% of the remaining patients classified as requiring surgery at a positive classification rate of 95%. Similar to the overall model, the number of imaging studies was the first splitting variable for non-FAP patients. However, a lower best cut-off level of 2.5 imaging studies was identified compared to 3.5 in the overall model. Because both groups contained ‘numbers of imaging studies’ as their first node, the DTA model suggests that the number of imaging studies was the most accurate variable on which to split patients who did vs. who did not require surgery. While it is not meant to be implicated as a causative factor, the number of imaging studies may reflect differences in healthcare access between the two groups, which influenced the subsequent need for surgeries.

## 4. Discussion

Estimated to affect nearly 3 million Americans, IBD is associated with significant morbidity and economic burden [17]. The current estimated annual cost of treating IBD is USD 6.3 billion in the US, and the average direct costs per patient per year is three-fold higher compared to non-IBD patients (USD 22,987 vs. USD 6956) [18]. Recently, biologic therapy has surpassed surgery and hospitalization as the primary driver of costs. In 2014, anti-tumor necrosis factor (TNF) agents accounted for 64.1% of healthcare costs in CD and 31.4% in UC. In contrast, hospitalizations and surgeries in CD patients accounted for 19% and <1% of their healthcare costs, respectively; for UC patients, hospitalizations and surgeries amounted to 23% and 1.4%, respectively [6]. In 2015, IBD patients requiring biologics accounted for USD 36,051 per patient per year costs attributed to outpatient IBD medications compared to <USD 5000 in IBD patients only requiring 5-ASA [19]. While contributing to a smaller proportion of costs in IBD, surgery remains a costly part of IBD management. Therefore, identifying patients at high-risk of increased HRU is important to improve outcomes and the economic impact of IBD. The rate of therapy maintenance may affect IBD-related surgery, which is one reason why we believe that access to PFA may improve outcomes for these patients. Escalating biologic therapy could enhance endoscopic remission, thus minimizing the surgery, hospitalizations and imaging required per patient.

Several prior studies have explored the relationship between socioeconomic status and HRU in IBD [11,12,13,14]. Because prior studies reported no associations between SES and disease phenotype, severity, and medication adherence [11,15], the association between HRU and SES in IBD may reflect disparities in healthcare access and, as a result, timely management of disease complications. In our current study, we study the impact of a FAP, intended to improve healthcare access, on outcomes and healthcare delivery in a cohort of indigent patients with IBD requiring biologic agents.

Studies in other chronic illnesses have demonstrated that minimizing healthcare disparities can have an immediate impact. In a cluster-randomized trial of black male barbershop patrons with uncontrolled hypertension, Victor et al. demonstrated hypertension screening and management delivered in barbershops resulted in much larger blood-pressure reductions compared to standard management by primary care practices [20]. In that study, the investigators improved healthcare access by bringing blood pressure screening to an at-risk patient population. At PMH, a FAP was implemented to help reduce financial burden and improve access to healthcare and medications for the indigent patient population in Dallas County, TX. For IBD, the FAP improves care by providing biologic agents at a significantly discounted co-pay, particularly anti-TNF agents, and removes the need for prior authorization. Similarly, to studies in other chronic diseases, we observed a beneficial impact of a FAP in the care of IBD patients. Specifically, we found that FAP patients were less likely to undergo surgery within 12 months after starting a biologic agent, even after adjusting for race and for variables relevant to disease severity such as IBD type, disease behavior, disease duration and prior IBD-related surgeries (OR: 0.28, 95% CI [0.08–0.91], *p* = 0.034).

To identify potential explanations for the difference in surgical rates, we found that the overall patient set (both FAP and non-FAP) had a higher cutoff value for imaging studies compared to non-FAP patients alone (3.5 vs. 2.5 studies) prior to undergoing surgery in the decision tree analysis. These findings suggest that the number of imaging studies was the best predictor of surgery among the two groups, and that the average patient who required surgery underwent more necessary imaging prior to surgery than non-FAP patients alone. This possibly suggests that the number of imaging studies is a manifestation of differences in healthcare system access between FAP and non-FAP patients, resulting in differing risks of surgery. In other words, it may be easier for FAP patients to obtain required imaging studies compared to non-FAP patients, due to lower burden from prior authorization or co-payments. We speculate that because FAP patients are better able to obtain necessary imaging studies to inform treatment adjustments following initiation of biologic therapy, they had better outcomes and less need for surgery. On the other hand, non-FAP patients potentially had more difficulties obtaining necessary imaging studies to monitor their disease until it was severe enough to require surgery. The finding of fewer imaging studies in the FAP vs. non-FAP group is, at face value, somewhat surprising. While not yet clear, it is also possible that the lower number of imaging studies in the FAP group could reflect the lower overall disease severity in this group, which created a less frequent need for imaging, compared to the non-FAP group, which may have received more imaging studies based on somewhat less well-controlled disease. We observed that FAP patients had a lower number of complex CD behaviors (50.7% for FAP versus 70% for non-FAP) while having similar average age and average body mass index, but a shorter disease duration than our non-FAP population. While additional studies are needed to validate this, it may suggest that FAP administration may reduce disease severity and the associated costs of managing treatment.

While our findings demonstrate benefits of improved healthcare access in an indigent IBD population, they also highlight the complexity of IBD management to prevent undesired outcomes such as surgery. Recent trends have demonstrated a decline in surgical rates in IBD, but this cannot be attributed to the advent of biologic agents alone. The decline of surgery rates in IBD more likely reflects the culmination of evolving practice patterns of early initiation of biologics, closer endoscopic and radiographic follow-up, stricter treatment targets, and better access to specialists [21,22]. Successfully managing each component of IBD care requires a significant amount of manpower and resources that are difficult to obtain even in a non-indigent population. Thus, our findings also underline the need for support and resources to provide quality care in order to improve outcomes and lessen the economic burden on the healthcare system.

Our study has a couple of notable strengths. To the best of our knowledge, this is the first study to evaluate the efficacy of an intervention for improving healthcare access in an indigent cohort of IBD patients. Additionally, the study was conducted in a closed healthcare system, so we were able to track most aspects of our patient’s care. Our study also provided data from a large sample size. Several limitations of our study include its retrospective nature and the lack of systematic documentation of validated clinical and endoscopic indices for measuring remission in progress notes and endoscopic reports, which could underestimate response rates. Another possible limitation of this study is that our data show a significantly lower proportion of FAP patients had previous IBD-related surgeries. There are several causes of this which could include more limited access to HRU; it is possible but unlikely that these patients were simply ‘healthier’, given their similar rates of CSFR. Additional studies which evaluate such patient characteristics are therefore warranted. Furthermore, this study reports a single center experience at a ‘safety-net’ hospital system in Dallas, TX, so the extent to which our findings may be generalizable to other indigent IBD populations in other regions of the US remains unclear. However, our findings provide a foundation to initiate planning for optimized healthcare in high-risk patient populations designed to improve outcomes and reduce healthcare costs in IBD.

## 5. Conclusions

In conclusion, we observed that IBD patients requiring biologic pharmacotherapy and enrolled in FAP were less likely to require surgery after initiating biologic therapy and underwent more necessary imaging studies prior to needing surgery, compared to IBD patients not enrolled in FAP. These findings suggest that programs designed to improve access to care can potentially improve healthcare delivery and outcomes in IBD patients from low socioeconomic backgrounds. Further studies are needed to identify additional interventions which address healthcare disparities in IBD to improve outcomes and its growing economic burden.

## Figures and Tables

**Table 1:** Cohort demographics and differences between IBD records with and without financial assistance program (n = number of unique biologic prescriptions).

	FAP Records (*n* = 185)	Non-FAP Records (*n* = 73)	*p*-Value
Mean Age (Standard deviation (SD))	39 (12.7)	39.2 (13.1)	0.916 (Bin. Log. Reg.)
Mean Body Mass Index (SD, kg/m^2^)	27.6 (6.8)	27.1 (7.7)	0.576 (Bin. Log. Reg.)
Current Smoker, *n* (%)	12 (11.3)	8 (20.0)	0.276 (Chi Square)
Mean Disease Duration (SD, years)	8.4 (8.2)	10.5 (9.9)	0.095 (Chi Square)
**Active Meds @time of biologic initiation, *n* (%)**			
Steroid	79 (42.7)	36 (49.3)	0.410 (Chi Square)
5-ASA	113 (61.1)	36 (49.3)	0.113 (Chi Square)
Immunomodulator	108 (58.4)	40 (54.8)	0.700 (Chi Square)
Biologic	70 (37.8)	23 (33.3)	0.418 (Chi Square)
Prior IBD-related surgery, *n* (%)	36 (19.6)	28 (38.4)	0.003 (Chi Square)
Biologic naïve, *n* (%)	107 (58.2)	43 (58.9)	1 (Chi Square)
**Healthcare access**			
Mean # of clinic visits (SD)	3.0 (1.4)	3.0 (1.3)	0.942 (Wilcoxon)
Days between Rx and initiation of biologic agent (SD)	31.6 (41.2)	42.8 (62.4)	0.104 (Wilcoxon)

**Table 2:** Cohort demographics and differences among IBD patients with and without financial assistance program (*n* = number of unique patients).

	FAP Patients (*n* = 140)	Non-FAP Patients (*n* = 64)	*p*-Value
**Female, *n* (%)**	59 (42.1)	38 (59.4)	0.032
**Race, *n* (%)**			**<0.001**
White	36 (25.7)	17 (26.6)	
Black	35 (25.0)	35 (54.7)	
Hispanic	64 (45.7)	9 (12.3)	
Asian	4 (2.9)	3 (4.7)	
Other	1 (0.7)	0	
**IBD Diagnosis, *n* (%)**			0.137
Ulcerative colitis	70 (50)	22 (34.4)	
Crohn’s disease	69 (49.3)	40 (62.5)	
IBD-Unclassified	4 (2.9)	2 (3.1)	
**Mean number of Biologics**	1.32 (0.55)	1.14 (0.47)	0.002 (Wilcoxon)
**Crohn’s Disease Location,** ***n* (% of CD patients)**			0.057
Small bowel	9 (13)	6 (15)	
Colonic	24 (34.8)	10 (25)	
Ileocolonic	37 (53.6)	22 (55)	
**Upper GI Involvement,** ***n* (% of CD patients)**	7 (15.6)	1 (3.3)	0.184
**Complex CD behavior,** ***n* (% of CD patients)**	**35 (50.7)**	**28 (70)**	**0.033**
**Perianal Crohn’s disease,** ***n* (% of CD patients)**	23 (33.3)	16 (40.0)	0.518
**Ulcerative colitis Location,** ***n* (% of UC patients)**			0.098
Proctitis	0	0	
Left-sided	14 (20)	9 (40.9)	
Pancolonic	57 (81.4)	13 (59.1)	

**Table 3:** Differences in outcomes and healthcare resource utilization between IBD records with and without financial assistance program (*n* = number of unique biologic prescriptions).

	FAP Records (*n* = 185)	Non-FAP Records (*n* = 73)	*p*-Value	Odds Ratio [95% CI]
**Clinical outcomes, *n* (%)**				
Clinical remission at 6 months	60 (38.2)	21 (34.4)	0.845	-
Clinical remission at 12 months	56 (45.2)	18 (36)	0.526	-
**Healthcare resource utilization**				
IBD-related Hospitalization, *n* (%)	64 (35.4)	30 (41.7)	0.590	-
IBD-related surgery, *n* (%)	10 (5.6)	12 (16.7)	0.034	0.28 [0.08–0.91] ^α^
Mean number of ED visits (SD)	0.63 (1.65)	1.03 (4.67)	0.781	-
Mean number of CT and MRI studies in 12 months (SD)	0.71 (0.96)	0.92 (1.33)	0.518	

^α^ Multivariable logistic regression analysis performed adjusting for age, gender, race, IBD diagnosis, prior IBD-related surgery, disease duration, and complex CD behavior.

## Data Availability

Data are available on request to authors.
